# Changes in pain scores and walking distance after transforaminal epidural steroid injection in patients with lumbar foraminal spinal stenosis

**DOI:** 10.1097/MD.0000000000034032

**Published:** 2023-06-23

**Authors:** Minsoo Kim, Jiwon Bak, Daehun Goh, Jangho Bae, Kiyoung Shin, Hee-Jeong Son, Jin Huh, Seong-Sik Kang, Byeongmun Hwang

**Affiliations:** a Department of Anesthesiology and Pain Medicine, Kangwon National University Hospital, School of Medicine, Kangwon National University, Chuncheon, Gangwon-do, Republic of Korea.

**Keywords:** low back pain, magnetic resonance imaging, spinal stenosis, steroids, walking

## Abstract

Transforaminal epidural steroid injections (TFESI) are widely used in patients with lumbar foraminal spinal stenosis. Previous studies have evaluated the effects of TFESI on lumbar foraminal spinal stenosis using only pain scores. However, no study has evaluated the effect of TFESI on pain scores and walking distance in patients with lumbar foraminal spinal stenosis. This study aimed to assess the effect of TFESI on pain scores and walking distance in patients with lumbar foraminal spinal stenosis stratified according to disease severity. This retrospective study reviewed the medical records of patients who received TFESI for lumbar foraminal spinal stenosis. A total of 128 patients were divided into the moderate and severe groups based on the extent of fat obliteration and the presence of nerve root compression. A significant decrease in the numeric rating scale (NRS) scores was observed in the moderate and severe groups compared with the corresponding baseline values 4 weeks after TFESI; however, the NRS pain scores were lower in the moderate group than those in the severe group. In addition, the proportion of patients who experienced pain reduction (≥50%) was higher in the moderate group than that in the severe group. The moderate and severe groups showed a significant increase in walking distance compared with the baseline values 4 weeks after the treatment. However, the walking distance values did not differ significantly between the moderate and severe groups. Furthermore, the degree of satisfaction was higher in the moderate group than that in the severe group. Lumbar TFESI may reduce pain scores and increase walking distance in patients with moderate or severe lumbar foraminal spinal stenosis. Patients with moderate foraminal stenosis had better pain relief outcomes than those with severe foraminal stenosis.

## 1. Introduction

Lumbar foraminal spinal stenosis is a disorder characterized by the narrowing of the bony exit of the nerve root^[1,2]^ that manifests as radicular pain in the lower extremities and impaired walking ability. Transforaminal epidural steroid injection (TFESI) reduces lower back or leg pain and enables daily activities by reducing the nerve root inflammation induced by mechanical compression of the narrowed foramen.^[3,4]^ Thus, TFESI has been increasingly used in patients with lumbar foraminal spinal stenosis.^[1,2]^

Previous studies have reported positive results regarding the role of TFESI in pain reduction in patients with lumbar foraminal spinal stenosis.^[5–7]^ TFESI provides a significant decrease in pain intensity and an improved walking distance in the third week compared with the baseline in patients with lumbar central spinal stenosis.^[8]^ Moreover, TFESI reduces radicular pain and improves walking tolerance in patients with degenerative lumbar spinal stenosis.^[9]^ However, research on the effect of TFESI on walking ability in patients with lumbar foraminal spinal stenosis is limited. Consequently, the effect of TFESI on impaired walking ability in patients with lumbar foraminal spinal stenosis remains unclear.

Pain and impaired walking are the main symptoms observed in patients with lumbar spinal stenosis. Hence, adequate assessment of walking ability and pain levels after TFESI in patients with lumbar spinal stenosis is paramount for evaluating treatment effectiveness. To the best of our knowledge, no previous study has investigated the effects of TFESI on pain scores and walking distance in patients stratified according to the severity of lumbar foraminal spinal stenosis. Therefore, this study aimed to assess the effect of TFESI on pain scores and walking distance in patients with moderate and severe lumbar foraminal spinal stenosis.

## 2. Methods

### 2.1. Study population

We performed a retrospective analysis of the data of patients who received TFESI for lumbar foraminal stenosis at the pain management practice center of Kangwon National University Hospital between July 2015 and December 2021. This study was approved by the institutional review board (No. KNUH-2022-07-006) and was conducted in accordance with the Declaration of Helsinki.

The inclusion criteria for the study were adults aged ≥40 years who had received TFESI for the management of lumbar foraminal stenosis presenting with ≥3-month history of symptomatic lumbar radicular pain, walking impairment due to lumbar foraminal stenosis with positive provocation factors, and moderate or severe foraminal stenosis in the lumbar spine MRI scan within 6 months before TFESI. Positive provocation factors included leg symptoms elicited or aggravated by walking but relieved by sitting down. The exclusion criteria were the presence of severe central stenosis or herniated intervertebral disc, spondylolisthesis in the lumbar region, previous lumbar spine or femur surgery, epidural corticosteroid injection within the preceding 6 months, or presence of any other disorders that could limit ambulation, such as severe osteoarthritis of the hip or knee, severe cardiovascular disease, chronic obstructive pulmonary disease, and neurologic deficits. In total, the medical records of 485 consecutive patients who had received TFESI for the management of lumbar foraminal stenosis and had a lumbar spine MRI scan available were reviewed. Among them, 128 patients who satisfied the inclusion criteria were selected after age (±2 years) matching. The participants were divided into 2 groups, the moderate and severe foraminal stenosis groups, with 64 patients in each group. The following information was collected by reviewing the medical records of the participants: medical history, pain scores, patient satisfaction scores, and intermittent claudication distance.

Lumbar foraminal stenosis was diagnosed based on medical history, including clinical symptoms and physical, neurological, and MRI findings. The diagnostic criteria for foraminal stenosis based on sagittal MRI used in this study were based on a study by Lee et al^[10]^ The “moderate” stenosis category referred to perineural fat obliteration in 4 directions without morphologic changes in both the vertical and transverse directions, whereas “severe” stenosis referred to nerve root collapse or morphologic change.^[10]^ A radiologist blinded to the other study data assessed the severity of foraminal stenosis and classified it as moderate or severe. The patients also received medications, including nonsteroidal anti-inflammatory drugs, muscle relaxants, neuromodulators (pregabalin and/or gabapentin), and limaprost.

Pain levels were assessed using the numeric rating scale (NRS), with scores ranging from 0 (no pain) to 10 (worst pain).^[8]^ A ≥50% reduction in the NRS pain score from the baseline indicated a therapeutic effect.^[5]^ The walking capacity of patients was expressed as walking distance,^[11–13]^ which was measured as the maximum distance that a person could walk on a walking track at their own pace without taking a break. Patients continuously walked until they were forced to stop or had to stop owing to the symptoms of neurogenic claudication. The minimum clinically important difference was estimated to be an improvement in walking distance of 30% or more from baseline.^[14]^ The NRS scores and walking distance were measured at baseline (before TFESI) and 4 weeks after TFESI to assess the treatment effects. The degree of patient satisfaction was assessed using the Roland 5-point patient satisfaction scale (0, 1, 2, 3, 4, and 5 points, representing absence of pain, mild pain, moderate pain, bad pain, very bad pain, and nearly unbearable pain, respectively) 4 weeks after TFESI.^[15]^ Patients with a Roland scale rating of 0 to 2 were considered successful responders with improvement.

### 2.2. Transforaminal epidural steroid injection

All patients in this study received lumbar TFESI at the most stenotic level on fluoroscopic imaging. All patients were placed in a prone position and supported with a pillow under the abdomen to reduce lumbar lordosis. The injection site was cleaned 3 times with 10% povidone-iodine solution and covered with sterile drapes. During TFESI, the fluoroscopy device was positioned at a 20° to 30° oblique angle and a 0° to 15° cranio-caudal angle to visualize the neural foramen. Subsequently, the injection site and subcutaneous tissue were infiltrated with local anesthetic (approximately 2 mL of 1% lidocaine), and a 25-gauge spinal needle was inserted into the subpedicular area under intermittent fluoroscopic visualization using the coaxial technique (directed to the 6 o’clock position). The needle position was confirmed using lateral imaging while approaching the epidural area. During the intervention, 1 to 2 mL of contrast dye was administered to confirm that the needle was in the epidural space in the posteroanterior and lateral images. After confirming the presence of epidural distribution without vascular distribution, a solution containing 5 mg dexamethasone, 2 mL saline, and 2 mL lidocaine hydrochloride (1%, preservative-free) was injected. The patients were referred to the observation room after the intervention and observed for 30 minutes to 1 hour, and patients with no complications were discharged. All patients returned to the clinic for treatment outcome assessment 4 weeks later. No other interventions were offered to the study patients; however, the patients continued therapy with non-opioid analgesics and pursued normal activities, including work.

### 2.3. Sample size

The primary outcome was pain reduction based on the NRS score. The secondary outcome was an improvement in walking distance. The sample size was determined based on previous assessments and studies.^[5,16]^ As the change in the NRS pain score was predicted as 50%, considering a 0.05 2-sided significance level, a power of 80%, an allocation ratio of 1:1, and an effect size of 0.5, 64 patients were included in each group.

### 2.4. Statistical analysis

Statistical analyses were performed using SPSS 25.0 (IBM Corporation, Armonk, NY). The data are presented as mean ± standard deviation or count (%), and a 95% confidence interval (CI) was added where appropriate. Among-group comparisons of the demographic and clinical characteristics were performed using Student *t* test. The changes in the parameters relative to the baseline values were analyzed using the paired *t* test within each group. The chi-square test was used to compare the differences in categorical variables between the groups. The assumption of normal distribution of the data was verified using Levene test. In all comparisons, *P* values < .05 were considered statistically significant.

## 3. Results

In total, 128 patients were included in this study and divided into the moderate and severe groups, with 64 patients in each group. Table 1 presents the baseline patient characteristics. The groups showed no significant differences in the demographic and clinical characteristics (Table 1). The data of continuous variables followed a normal distribution.

**Table 1:** Clinical and demographic characteristics of the study population.

Characteristic	Severity of lumbar foraminal stenosis		
	Moderate group (n = 64)	Severe group (n = 64)	*P* values
Age (yr)	67.4 ± 8.9	68.2 ± 8.8	.843
Sex (male, %)	28 (44%)	32 (50%)	.715
BMI (kg/m^2^)	23.3 ± 2.5	23.5 ± 2.6	.512
Duration of symptoms (y)	4.2 ± 4.0	4.9 ± 4.6	.453
Medications			
NSAIDs	41 (64%)	39 (61%)	.855
Muscle relaxants	33 (52%)	25 (39%)	.214
Neuromodulators	11 (17%)	9 (14%)	.808
Limaprost	52 (81%)	54 (84%)	.815
Injection sites			
L4–L5	14 (22%)	10 (16%)	.497
L5–S1	44 (69%)	46 (72%)	.847
S1	6 (9%)	8 (13%)	.777

Values are presented as mean ± standard deviation or count (%). The “moderate” and “severe” groups included patients with moderate and severe lumbar foraminal stenosis, respectively, who received transforaminal epidural steroid injections. BMI = body mass index, NSAID = nonsteroidal anti-inflammatory drug. Differences are considered statistically significant at *P* values of <.05.

The mean baseline walking distance values in the moderate and severe groups were 563 ± 265 m and 528 ± 289 m, respectively, with no significant differences. However, 4 weeks after TFESI, the mean walking distance values in the moderate and severe groups were 660 ± 317 and 595 ± 327 m, respectively. The moderate and severe groups showed a significant increase in walking distance compared with the baseline values (*P* < .001; Table 2) 4 weeks after treatment. The proportion of patients who experienced at least 30% improvement in walking distance in the moderate and severe groups was 27% (95% CI = 16–37) and 22% (95% CI = 12–32), respectively. However, the mean walking distance values did not differ significantly between the groups 4 weeks after TFESI (*P* = .251; Table 2).

**Table 2:** Changes in walking distance.

	Walking distance (meters)		*P* value†
	Moderate group	Severe group	
Baseline	563 ± 265	528 ± 289	.475
4 wk after TFESI	660 ± 317	595 ± 327	.251
*P* value‡	<.001*	<.001*	.680
≥30% improvement in walking distance§ (%), [95% CI]	17 (27%)	14 (22%)	
	[16–37]	[12–32]	

The “moderate” and “severe” groups included patients with moderate and severe lumbar foraminal stenosis, respectively, who received transforaminal epidural steroid injections. Values are presented as mean ± standard deviation.

† Student *t* test (for comparison of mean values between groups;

* *P* values of <.05 are considered significant).

‡ Paired *t* test (for comparison of intragroup mean values; *P values of <.05 are considered significant).

§ Improvement in walking distance (≥30%) refers to the number (%, [95% CI]) of patients who achieved at least 30% improvement in walking distance at 4 wk after TFESI. Chi-square test (for comparison of mean values between groups; *P values of <.05 are considered significant).

CI = confidence interval, TFESI = transforaminal epidural steroid injection.

The baseline means NRS scores in the moderate and severe groups were 6.3 ± 0.8 and 6.1 ± 0.9 points, respectively, with no significant differences between the groups. However, 4 weeks after TFESI, the mean NRS scores in the moderate and severe groups decreased to 3.7 ± 1.7 and 4.8 ± 1.8 points, respectively. The NRS pain scores at 4 weeks after TFESI were lower in the moderate and severe groups than the baseline values (*P* < .001; Table 2). In addition, 4 weeks after TFESI, the NRS pain scores in the moderate group were lower than those in the severe group (*P* < .001; Table 3). The proportion of patients who experienced pain reduction (≥50%) in the moderate and severe groups was 66% (95% CI = 54–77) and 39% (95% CI = 27–51), respectively. The proportion of patients who experienced pain reduction (≥50%) in the moderate group was higher than that in the severe group (*P* = .005; Table 3).

**Table 3:** Changes in pain intensity evaluated using the numeric rating scale (NRS) scores.

	Numeric rating scale score (points)		*P* value†
	Moderate group	Severe group	
Baseline	6.3 ± 0.8	6.1 ± 0.9	.243
4 wk after TFESI	3.7 ± 1.7	4.8 ± 1.8	<.001*
*P* value‡	<.001*	<.001*	
≥50% pain reduction§	42 (66%)	25 (39%)	.005*
(%), [95% CI]	[54–77]	[27–51]	

The “moderate” and “severe” groups included patients with moderate and severe lumbar foraminal stenosis, respectively, who received transforaminal epidural steroid injections. Values are presented as mean ± standard deviation.

CI = confidence interval, TFESI = transforaminal epidural steroid injection.

† Student *t* test (for comparison of mean values between groups;

* *P* values of <.05 are considered significant).

‡ Paired *t* test (for comparison of intragroup mean values; *P values of <.05 are considered significant).

§ Pain reduction (≥50%) refers to the number (%, [95% CI]) of patients who showed a pain reduction of ≥50% at 4 wk after TFESI. Chi-square test (for comparison of mean values between groups; *P values of <.05 are considered significant).

Furthermore, 4 weeks after TFESI, the proportion of patients with an improvement in the degree of satisfaction in the moderate and severe groups was 59% and 33%, respectively (Table 4). The proportion of patients with an improvement in the degree of satisfaction was higher in the moderate group than that in the severe group (*P* = .005; Table 4).

**Table 4:** Patient satisfaction ratings.

Variable	Moderate group (n = 64)	Severe group (n = 64)	*P* value
0−1	14 (22%)	6 (9%)	
2	24 (38%)	15 (23%)	
3	18 (28%)	32 (50%)	
4−5	8 (13%)	11 (17%)	
Improvement	38 (59%)	21 (33%)	.005*

Values represent counts (%). The “moderate” and “severe” groups included patients with moderate and severe lumbar foraminal stenosis, respectively, who received transforaminal epidural steroid injections. Patients with a Roland scale rating of 0–2 were considered as successful responders with improvement. The chi-square test (for comparison of mean values between groups;

* *P* values of <.05 are considered significant).

## 4. Discussion

In this study, we investigated the effects of TFESI on pain scores, walking distance, and satisfaction levels of patients with moderate and severe lumbar foraminal spinal stenosis classified according to the degree of foraminal stenosis. The moderate and severe groups showed a significant decrease in pain scores compared with the baseline values 4 weeks after TFESI. However, the moderate group had lower pain scores and a higher proportion of patients with a pain reduction of ≥50% from the baseline compared with the severe group. Both groups showed a significant increase in walking distance 4 weeks after TFESI, with no significant difference between the moderate and severe groups. In addition, the moderate group had a higher level of satisfaction.

Spinal stenosis may lead to continuous mechanical stimulation of the nerves and repeated inflammation of the nerve root when the condition persists. TFESI, including steroids and local anesthetics, inhibits prostaglandin synthesis, stabilizes cellular membranes, suppresses immune responses, increases neuronal blood flow, and washes out inflammatory mediators.^[17,18]^ Moreover, TFESI provides a clinically significant improvement in radiculopathy and neurogenic claudication caused by lumbar spinal stenosis.^[19,20]^ Notably, TFESI is more effective in treating foraminal stenosis than other injection procedures, including interlaminar and caudal injections, due to the higher steroid density in the dorsal root ganglion.^[21–23]^

Evidence for the use of TFESI for radicular pain caused by foraminal stenosis is limited compared with that for discogenic radicular pain.^[3]^ Several studies have evaluated the effects of TFESI in patients with lumbar foraminal stenoses.^[5,6]^ Park et al reported that the mean pain scores decreased by −3.0 4 weeks after TFESI compared with that before its administration.^[5]^ Chang et al reported that the mean pain scores decreased by −3.3 and −2.5 in the mild to moderate and severe groups, respectively, a month after TFESI.^[6]^ In the present study, the mean pain scores decreased by −2.6 and −1.3 in the moderate and severe groups, respectively, 4 weeks after receiving TFESI. The overall change in pain score after TFESI was 2.0. Similar to the findings by Chang et al, the reduction in pain scores was more pronounced in the group with a moderate degree of lumbar foraminal stenosis than in the group with a severe degree of lumbar foraminal stenosis.^[6]^ In contrast, Yuce et al and Donohue et al reported a decrease in pain scores in patients who underwent TFESI for the treatment of radicular low back pain; however, making a direct comparison with the findings of the present study is difficult.^[1,4]^

TFESI is an effective treatment modality for pain relief and functional improvement in patients with foraminal stenosis.^[4]^ Olguner et al reported that 64% of the patients with foraminal stenosis experienced pain relief (pain score reduction of ≥50%).^[24]^ Park et al reported that 70% of patients with lumbar foraminal stenosis showed ≥50% reduction in pain at 4 weeks after TFESI.^[5]^ Donohue et al reported that 75% of patients showed functional improvement in activities of daily living.^[4]^ In the present study, the group with a moderate degree of lumbar foraminal stenosis showed a ≥50% reduction in pain in 66% of patients 4 weeks after TFESI, similar to the results of previous studies.^[4,5,24]^ In addition, successful pain relief in the moderate-degree group was higher than that in the severe degree of lumbar foraminal stenosis.

However, there is insufficient evidence suggesting that epidural injections improve walking ability.^[25]^ Recent studies have reported that walking distance increases in patients with lumbar central spinal stenosis after interlaminar epidural steroid injection.^[8,16]^ Botwin et al reported that 75% of patients had successful outcomes (at least ≥50% reduction in pain scores), and 64% of patients who received TFESI for lumbar stenosis had improved walking tolerance.^[9]^ In the present study, the walking distance increased in the moderate and severe groups of patients with lumbar foraminal spinal stenosis after receiving TFESI. This finding suggests that TFESI has a positive effect on walking ability in patients with lumbar foraminal stenosis. The reason that there was no difference in the walking distance in the moderate and severe groups after TFESI may be because the patients had similar walking distances at baseline, independent of their severity of foraminal stenosis. In the present study, 52% of patients showed a reduction of ≥50% in pain score, and 24% of patients showed an improvement of ≥30% in walking distance. Lesser number of patients showed clinically important improvement in walking distance compared with the number of patients with pain relief, suggesting that TFESI should not be recommended as a treatment option for impaired walking ability in patients with moderate and severe lumbar foraminal spinal stenosis.

The overall success rate of TFESI is high; however, most benefits are observed in the immediate and early post-injection periods.^[26]^ In a study by Park et al that used a patient satisfaction scale, 67.5% of patients showed pain relief 2 weeks after receiving TFESI.^[7]^ Tafazal et al reported that the proportion of patients who showed improvements (excellent and good) 3 months after receiving TFESI was 42% among patients with foraminal stenosis.^[27]^ In the present study, the overall proportion of patients who showed improvements 4 weeks after receiving TFESI was 46% (the group with a moderate grade of lumbar foraminal stenosis: 59%). Moreover, TFESI reduced the pain scores and increased the walking distance in patients with lumbar foraminal stenosis, and the degree of patient satisfaction was relatively high. However, patients with moderate foraminal stenosis had better outcomes than those with severe foraminal stenosis. Based on these findings, pain management physicians should inform patients with severe foraminal stenosis that the effectiveness of TFESI in their condition may be unsatisfactory.

This study had some limitations. First, this was a single-center observational study. However, we used restrictive eligibility criteria to reduce selection bias and included patients with similar age ranges, lifestyles, and baseline pain scores. Second, the patients received different types of oral medications, which may have affected the therapeutic effects of TFESI. However, the medications received by these patients are routinely prescribed for lumbar spinal stenosis. Third, this study assessed the short-term outcomes. However, to the best of our knowledge, this study is the first to investigate the effects of TFESI on pain scores and walking distance in patients stratified according to the severity of lumbar foraminal stenosis. Prospective randomized controlled trials are required to investigate the long-term effects of TFESI on pain scores and walking distances in patients with lumbar foraminal stenosis.

## 5. Conclusion

In conclusion, lumbar TFESI may reduce pain scores and increase walking distance in patients with moderate or severe lumbar foraminal spinal stenosis. Moreover, this study showed that patients with moderate foraminal stenosis had better outcomes in terms of pain relief than those with severe foraminal stenosis.

## Author contributions

**Conceptualization:** Minsoo Kim, Byeongmun Hwang.

**Data curation:** Daehun Goh, jangho Bae, Kiyoung Shin.

**Formal analysis:** Hee-Jeong Son, Jin Huh, Seong-Sik Kang.

**Investigation:** Daehun Goh, Byeongmun Hwang.

**Resources:** Minsoo Kim, Jiwon Bak, Daehun Goh, Byeongmun Hwang.

**Supervision:** Byeongmun Hwang.

**Writing – original draft:** Byeongmun Hwang.

**Writing – review & editing:** Byeongmun Hwang.
